# Hexavalent chromium induces apoptosis in male somatic and spermatogonial stem cells via redox imbalance

**DOI:** 10.1038/srep13921

**Published:** 2015-09-10

**Authors:** Joydeep Das, Min-Hee Kang, Eunsu Kim, Deug-Nam Kwon, Yun-Jung Choi, Jin-Hoi Kim

**Affiliations:** 1 Department of Animal Biotechnology, College of Animal Bioscience and Biotechnology/Animal Resources Research Center, Konkuk University, Seoul 143-701, South Korea

## Abstract

Hexavalent chromium [Cr(VI)], an environmental toxicant, causes severe male reproductive abnormalities. However, the actual mechanisms of toxicity are not clearly understood and have not been studied in detail. The present *in vitro* study aimed to investigate the mechanism of reproductive toxicity of Cr(VI) in male somatic cells (mouse TM3 Leydig cells and TM4 Sertoli cells) and spermatogonial stem cells (SSCs) because damage to or dysfunction of these cells can directly affect spermatogenesis, resulting in male infertility. Cr(VI) by inducing oxidative stress was cytotoxic to both male somatic cells and SSCs in a dose-dependent manner, and induced mitochondria-dependent apoptosis. Although the mechanism of Cr(VI)-induced cytotoxicity was similar in both somatic cells, the differences in sensitivity of TM3 and TM4 cells to Cr(VI) could be attributed, at least in part, to cell-specific regulation of P-AKT1, P-ERK1/2, and P-P53 proteins. Cr(VI) affected the differentiation and self-renewal mechanisms of SSCs, disrupted steroidogenesis in TM3 cells, while in TM4 cells, the expression of tight junction signaling and cell receptor molecules was affected as well as the secretory functions were impaired. In conclusion, our results show that Cr(VI) is cytotoxic and impairs the physiological functions of male somatic cells and SSCs.

Chromium (Cr) is a naturally occurring element that exists in a variety of oxidation states (−2 to +6). Among the ionic forms of Cr, hexavalent chromium [Cr(VI)], the most toxic form, can readily cross cellular membranes via nonspecific anion transporters^1^. After entering the cell, Cr(VI) is reduced to produce reactive intermediates, including Cr(V), Cr(IV), Cr(III), and reactive oxygen species (ROS)^2^. These species can cause DNA strand breaks, base modifications, and lipid peroxidation, thereby disrupting cellular integrity and inducing toxic, as well as mutagenic effects^3^. Cr(VI) is used in more than 50 different industries worldwide in a variety of applications, including pigment and textile production, leather tanneries, wood processing, chrome plating, metallurgical and chemical industries, stainless steel factories, welding, cement manufacturing factories, ceramic, glass, and photographic industries, catalytic converter production for automobiles, heat resistance, and as an anti-rust agent in cooling plants^4^,^5^. The increased use by industries, coupled with improper disposal of Cr(VI) waste, has resulted in an increase in the levels of Cr(VI) in soil, water, and air, leading to environmental pollution^6^,^7^,^8^,^9^. It is estimated that approximately half a million workers in the United States and several million workers worldwide have been exposed to Cr(VI) (via inhalation and skin contact)^9^.

Environmental or occupational exposure to Cr(VI) results in an increased risk of asthma, nasal septum lesions, skin ulcerations, and cancers of the respiratory system^9^. Cr(VI) is also known to cause cytotoxic, genotoxic, immunotoxic, and carcinogenic effects in both humans and laboratory animals^5^,^10^,^11^, as well as allergic dermatitis and reproductive toxicity^12^,^13^,^14^. In the welding industry, workers exposed to Cr(VI) have an increased risk of poor semen quality and sperm abnormalities that lead to infertility or cause developmental problems in children^15^. An increase in spermatozoa with abnormalities and a decrease in sperm count have also been reported in Cr-treated/exposed mice, rats, rabbits, and bonnet monkeys^13^,^14^,^16^,^17^.

Although Cr(VI) is known to affect male reproductive health, there is limited scientific data concerning the toxicity and there are no appropriate *in vitro* models to clearly understand the possible cytotoxic effects, including oxidative stress and apoptosis. In the present study, we investigated the mechanism underlying the toxic effects of Cr(VI) in male somatic and spermatogonial stem cells (SSCs). Leydig cells are somatic cells adjacent to the seminiferous tubules that produce the primary androgen, testosterone, an important hormone for the maturation of sperm. Sertoli cells are located in the convoluted seminiferous tubules and are responsible for supporting/promoting the development of germ cells. They also form the blood–testis barrier and provide physical support to SSCs, which are situated on the basement membrane of the seminiferous tubules, to form the stem cell niche. SSCs represent a self-renewing population of spermatogonia and support spermatogenesis by continuous division throughout the life of the male. Thus, damage to or dysfunction of the Leydig or Sertoli cells, and/or SSCs can have adverse effects on spermatogenesis and the production of sperm.

The objectives of the present *in vitro* study were to: (i) determine the cytotoxic effects of Cr(VI) on mouse TM3 cells (a well-known mouse Leydig cell line), mouse TM4 cells (a well-known mouse Sertoli cell line), and mouse SSCs; (ii) evaluate the effects of Cr(VI) on oxidative stress; (iii) assess the effects of Cr(VI) on apoptotic signaling mechanisms; (iv) understand the role of Cr(VI) in cell proliferation/self-renewal mechanisms of SSCs; and (v) explore the effects of Cr(VI) on the physiological functions of TM3 and TM4 cells.

## Results

### Cr(VI) induces apoptotic cell death in male somatic cells and SSCs

**Figure 1:**
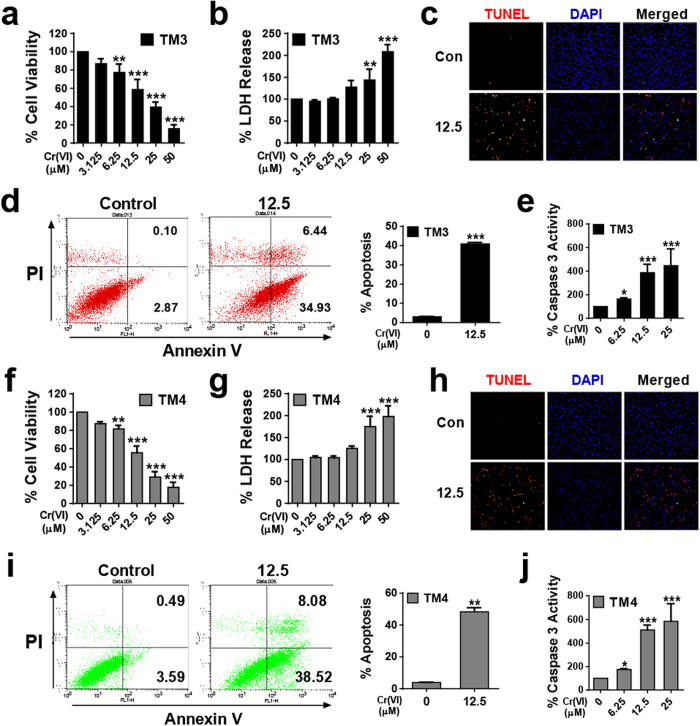
Cytotoxicity of Cr(VI) in TM3 and TM4 cells. (**a**,**f**) Cell viability relative to the control (100%) in mouse TM3 and TM4 cells, respectively, exposed to different concentrations of Cr(VI) in μM for 24 h. Cell viability was measured using the Cell Counting Kit-8 (CCK-8). (**b**,**g**) LDH release in TM3 and TM4 cells, respectively, treated with different concentrations of Cr(VI) in μM for 24 h. (**c**,**h**) Representative fluorescent images of TUNEL-positive (red) TM3 and TM4 cells, respectively, after exposure to 12.5 μM Cr(VI) for 24 h. Cell nuclei were counterstained with 4′, 6-diamidino-2-phenylindole (DAPI, blue). Magnification 100X. (**d**,**i**) Apoptosis (dot plot distribution and % apoptosis calculations) of TM3 and TM4 cells, respectively, measured by Annexin V/PI assay after exposure to 12.5 μM Cr(VI) for 24 h. (**e**,**j**) % Caspase 3 enzyme activity relative to the control (100%) in the mouse TM3 and TM4 cells, respectively, exposed to different concentrations of Cr(VI) in μM for 24 h. All values are expressed as mean ± SEM. (*n = 3*). *P < 0.05, **P < 0.01, and ***P < 0.001 compared to the control.

**Figure 2:**
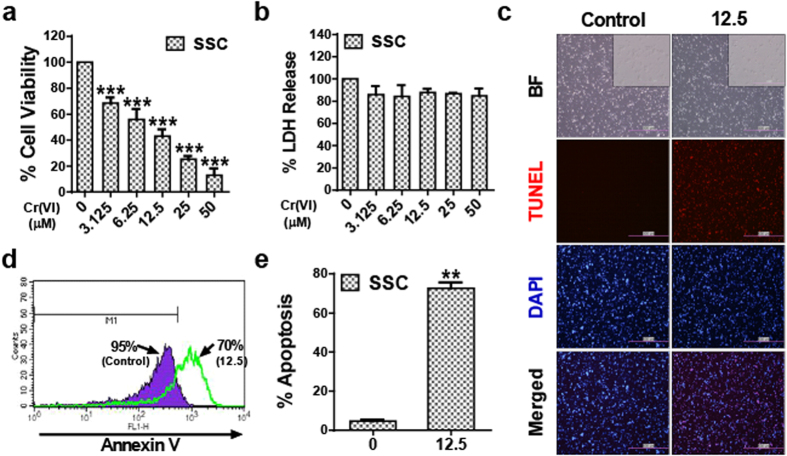
Cytotoxicity of Cr(VI) in SSCs. (**a**) Cell viability relative to the control (100%) in mouse SSCs exposed to different concentrations of Cr(VI) in μM for 24 h. Cell viability was measured using the Cell Counting Kit-8 (CCK-8). (**b**) LDH release in SSCs treated with different concentrations of Cr(VI) in μM for 24 h. (**c**) Representative bright field and fluorescent images of TUNEL-positive (red) SSCs after exposure to 12.5 μM Cr(VI) for 24 h. Cell nuclei were counterstained with 4′,6-diamidino-2-phenylindole (DAPI, blue). Magnification 100X. (**d**) Apoptosis in SSCs detected by Annexin V after exposure to 12.5 μM Cr(VI) for 24 h and measured by flow cytometry. M1 represents the population of non-apoptotic cells. (**e**) % of apoptosis in SSCs measured by Annexin V/PI assay after exposure to 12.5 μM Cr(VI) for 24 h. All values are expressed as mean ± SEM. (*n* = 3). *P < 0.05, **P < 0.01 and ***P < 0.001 compared to the control.

To determine the cytotoxic effect of Cr(VI), cell viability and lactate dehydrogenase (LDH) release from the cells were measured after culturing the cells in the presence of different concentrations of Cr(VI) (0, 3.125, 6.25, 12.5, 25, and 50 μM) for 24 h. As shown in Fig. 1, Cr(VI) decreased the cell viability and increased the release of LDH into the culture medium in a dose-dependent manner for both TM3 and TM4 cells. The IC_50_ value for the somatic cells was measured as 12.5 μM. In SSCs, Cr(VI) decreased the cell viability in a dose-dependent manner without LDH release (Fig. 2). This indicated that Cr(VI) interfered with cell metabolism, but did not disrupt the membranes of the SSCs. The IC_50_ value for the SSCs was measured as 6.25 μM. Flow cytometry analysis using annexin-V/PI, terminal deoxynucleotidyl transferase (TdT)-mediated dUTP nick end labeling (TUNEL), and caspase 3 enzyme activation assays showed that the mode of death induced by Cr(VI) in both TM3 and TM4 cells was apoptosis. Treatment with 12.5 μM Cr(VI) increased the percentage of apoptotic cells (analyzed by flow cytometry) and TUNEL-positive cells compared to those observed after treatment with control (Fig. 1). In addition, Cr(VI) increased caspase 3 enzyme activity in both cell types in a dose-dependent manner (Fig. 1). In the SSCs, treatment with 12.5 μM Cr(VI) increased the percentage of annexin-V-positive and TUNEL-positive cells compared to that in the control, indicative of apoptosis (Fig. 2). These results show that Cr(VI) caused apoptosis in all the cells and the relative IC_50_ results suggest that SSCs are more sensitive to Cr(VI) than the somatic cells.

### Cr(VI) induces oxidative stress and disrupts mitochondrial membrane potential in male somatic cells and SSCs

**Figure 3:**
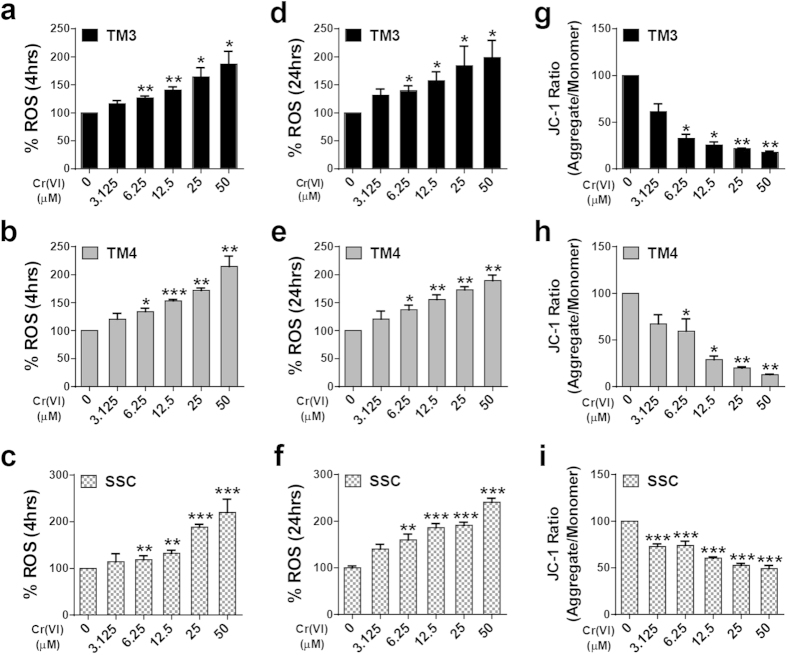
Effects of Cr(VI) on ROS and MMP in somatic cells and SSCs. (**a–c**) ROS production in TM3, TM4, and SSCs respectively after treatment with different concentrations of Cr(VI) for 4 h. (**d–f**) ROS production in TM3, TM4, and SSCs after treatment with different concentrations of Cr(VI) for 24 h. ROS production in cells was measured by using a cationic fluorescent dye, 2′, 7′-dichlorodihydrofluorescein diacetate (H2-DCFDA). (**g**–**i**) MMP (as a ratio of JC1 aggregate to JC1 monomer) in TM3, TM4, and SSCs after treatment with different concentrations of Cr(VI) for 24 h. All values are expressed as mean ± SEM. (*n = 3*). *P < 0.05, **P < 0.01 and ***P < 0.001 compared to the control.

We measured the formation of reactive oxygen species (ROS) and loss of mitochondrial membrane potential (MMP) in cells exposed to Cr(VI). The production of intracellular ROS increased in a dose-dependent manner in all cell types after 4 h incubation with Cr(VI) and was maintained up to 24 h (Fig. 3). In contrast, the MMP decreased in a dose-dependent manner, after 24 h exposure to Cr(VI) (Fig. 3). Pre-treatment (5 mM, 1 h) with the ROS inhibitor, N-acetyl-L-cysteine (NAC), significantly counteracted the effects of Cr(VI) on cell viability, ROS formation, and MMP in all the cell types (Supplementary Figs 1,2 and 3). These results indicated that the Cr(VI)-induced cytotoxicity in male somatic cells and SSCs was mediated through oxidative stress and subsequent mitochondrial damage.

### Cr(VI) alters mRNA expression of antioxidant enzymes in male somatic cells and SSCs

**Figure 4:**
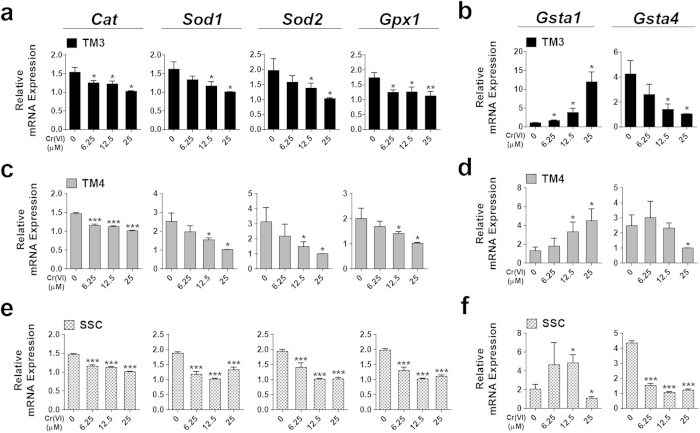
Effects of Cr(VI) on mRNA expression of antioxidant enzymes (*Cat, Sod1, Sod2, Gpx1, Gsta1*, and *Gsta4*) in somatic cells and SSCs. (**a**,**b**) Relative mRNA expression was analyzed by qRT-PCR in TM3 cells after treatment with different concentrations of Cr(VI) for 24 h. (**c**,**d**) Relative mRNA expression was analyzed by qRT-PCR in TM4 cells after treatment with different concentrations of Cr(VI) for 24 h. (**e**,**f**) Relative mRNA expression was analyzed by qRT-PCR in SSCs after treatment with different concentrations of Cr(VI) for 24 h. All values are expressed as mean ± SEM. (*n = 3*). *P < 0.05, **P < 0.01 and ***P < 0.001 compared to the control.

Figure 4 shows the changes in the expression levels of antioxidant genes as measured by quantitative reverse transcription-polymerase chain reaction (qRT-PCR), in all the cell types exposed to increasing doses of Cr(VI). Cr(VI) decreased the mRNA expression levels of catalase (*Cat*), superoxide dismutase 1(*Sod1*), superoxide dismutase 2 (*Sod2*), glutathione peroxidase 1 (*Gpx1*), and glutathione S-transferase a4 (*Gsta4*) in a dose-dependent manner in all the cells compared to the values in the relevant control. However, there were some cell type-specific differences in the responses. In the somatic cells, Cr(VI) increased the mRNA expression of glutathione S-transferase a1 (*Gsta1*) in a dose-dependent manner. In the SSCs, expression of *Gsta1* increased with lower doses of Cr(VI), but decreased with a higher dose. Our results show that exposure to Cr(VI) disrupted cellular antioxidant defense mechanisms, leading to increased oxidative stress.

### Cr(VI) induces intrinsic apoptotic pathways via regulation of AKT1, P53, and MAPK family proteins in male somatic cells

**Figure 5:**
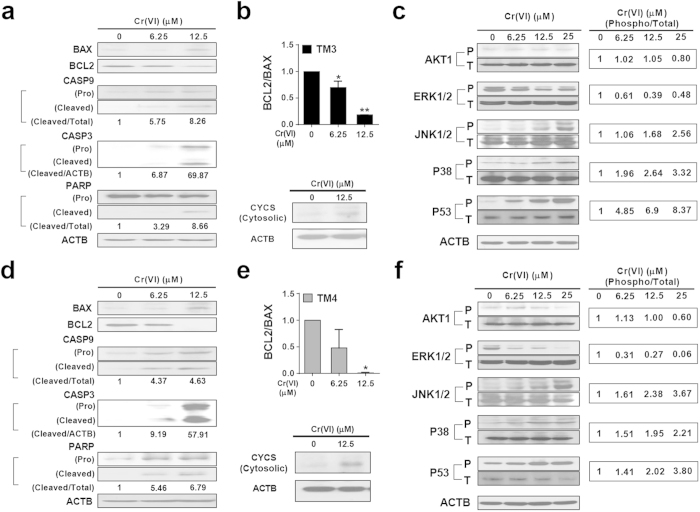
Effects of Cr(VI) on pro-apoptotic and pro-survival signaling pathways in TM3 and TM4 cells. (**a**,**b**) Western blot analysis of mitochondria-dependent apoptotic pathways in TM3 cells after treatment with different concentrations of Cr(VI) for 24 h. (**c**) Western blot analysis of phospho and total AKT1, MAPK and P53 proteins in TM3 cells after treatment with different concentrations of Cr(VI) for 24 h. (**d**–**e**) Western blot analysis of mitochondria-dependent apoptotic pathways in TM4 cells after treatment with different concentrations of Cr(VI) for 24 h. (**f**) Western blot analysis of phospho and total AKT1, MAPK and P53 proteins in TM4 cells after treatment with different concentrations of Cr(VI) for 24 h. BAX, BCL2, CASP9, CASP3, PARP, AKT1, ERK 1/2, JNK 1/2, P38, and P53 proteins were analyzed in the whole cell protein lysate. CYCS was analyzed in the cytosolic fraction. For the BCL2/BAX ratio, values are expressed as mean ± S.E.M. (*n = 3*). *P < 0.05, **P < 0.01 and ***P < 0.001 compared to the control. Densitometric analyses were carried out using Image J software.

Western blot analyses were performed to investigate the underlying mechanisms of the pro-apoptotic role of Cr(VI) in male somatic cells. Cr(VI) exposure for 24 h decreased the BCL2/BAX protein ratio and increased the expression of cleaved-caspase 9 (CASP9), caspase 3 (CASP3), and poly (ADP-ribose) polymerase (PARP) proteins in a dose-dependent manner (Fig. 5). Cr(VI) exposure, at a concentration of 12.5 μM for 24 h, increased the expression of cytosolic cytochrome c (CYCS, somatic) (Fig. 5). The levels of endogenous pro and cleaved-CASP3 were negligible in control (untreated) cells, but dose-dependently increased in both TM3 and TM4 cells after exposure to Cr(VI). Since pro-CASP3 is inactive and the cleaved-CASP3 is responsible for most of the catalytic activity of CASP3, we compared the levels of cleaved-CASP3 to those of beta actin (ACTB). The evidence for the dose-dependent increase of cleaved-CASP3 (Fig. 5) observed in the western blots was supported by the increase of caspase 3 enzyme activity (Fig 1e,j). We also investigated the extrinsic apoptotic pathways in the somatic cells by measuring the gene expression of fas (*Fas*), fas ligand (*Fasl*) and caspase 8 (*Casp8*). Exposure to Cr(VI) for 24 h increased the expression of *Fasl* mRNA but decreased the expression of *Fas* and *Casp8* (Supplementary Fig 4) indicating that Cr(VI) induced intrinsic (mitochondria-dependent) apoptosis in male somatic cells.

We examined the alterations in the expression of other proteins related to either cell survival or death in response to cellular stress. At concentrations up to 12.5 μM, Cr(VI) did not affect the ratio of P-AKT1/T-AKT1 protein (also known as protein kinase B) (Fig. 5). However, at a concentration of 25 μM Cr(VI), there was a 20% and 40% reduction in the expression of P-AKT1/T-AKT1 protein in TM3 and TM4 cells, respectively, compared to the corresponding values in the relevant control group. The expression of the protein ratios P-JNK1/2 to T-JNK1/2 (c-Jun N-terminal kinases) and P-P38/T-P38 (P38 mitogen-activated protein kinases) protein increased after exposure to Cr(VI) in a dose-dependent manner in somatic cells (Fig. 5). In TM3 cells, the relative change of P-P38 was more pronounced than P-JNK1/2, but this order was reversed in TM4 cells. In contrast, the expression of the protein ratios P-ERK1/2 to T-ERK1/2 (extracellular-signal-regulated kinases) decreased. A concentration of 25 μM Cr(VI) reduced P-ERK1/2 expression by two-fold in TM3 cells, but reduced it by a factor of 15 in TM4 cells compared to values in the relevant control. Cr(VI) also increased the expression of P-P53/T-P53 (transformation related protein 53) protein in a dose-dependent manner. A concentration of 25 μM Cr(VI), increased the expression of P-P53 expression by 8.4-fold in TM3 cells compared to a 3.8-fold increase in TM4 cells (Fig. 5). These results indicate that after exposure to Cr(VI), the P-AKT1, P-ERK1/2, and P-P53 proteins were regulated differently in TM3 and TM4 cells.

### Cr(VI) induces intrinsic apoptosis and disrupts the glial cell line-derived neurotrophic factor (GDNF) signaling pathways in SSCs

**Figure 6:**
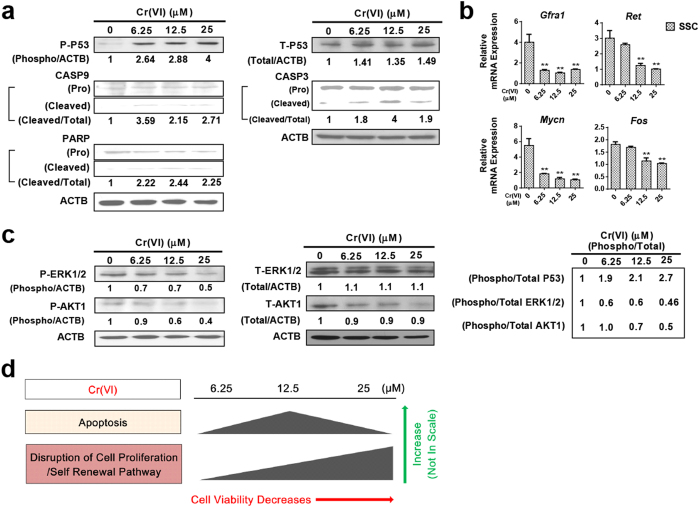
Effects of Cr(VI) on pro-apoptotic and GDNF signaling pathways in SSCs. (**a**) Western blot analysis of P53, CASP9, CASP3 and PARP proteins in SSCs after treatment with different concentrations of Cr(VI) for 24 h. (**b**) Relative mRNA expression of *Gfra1, Ret, Mycn* and *Fos* was analyzed by qRT-PCR in SSCs after treatment with different concentrations of Cr(VI) for 24 h. All values are expressed as mean ± S.E.M. (*n* = 3). *P < 0.05, **P < 0.01 and ***P < 0.001 compared to the control. (**c**) Western blot analysis of ERK 1/2 and AKT1 proteins in SSCs after treatment with different concentrations of Cr(VI) for 24 h. Densitometric analyses were carried out using Image J software. (**d**) Schematic diagram of Cr(VI) induced cytotoxicity in SSCs.

To further examine the cytotoxic effects of Cr(VI) on SSCs, we investigated the involvement of the intrinsic apoptotic pathway. Exposure to Cr(VI) dose-dependently increased the expression of the P-P53 protein (Fig. 6) and also increased the levels of cleaved CASP9, CASP3, and PARP proteins (Fig. 6). Considering the mitochondria-independent apoptosis pathway in SSCs, although, Cr(VI) increased the expression of *Fasl* mRNA, the expression of *Fas* and *Casp8* decreased (Supplementary Fig 4). In addition, while concentrations of Cr(VI) up to 12.5 μM increased apoptosis as evident from the dose-dependent cleavage of CASP3 and PARP proteins, higher concentrations (25 μM) decreased apoptosis, shown by the decreased cleavage of CASP3 and PARP proteins. We therefore hypothesized that some additional factors, apart from apoptosis, could bear responsibility for the observed loss of cell viability. Since the GDNF signaling pathway controls the self-renewal/maintenance of SSCs via AKT1–*Mycn* signaling mechanisms^18^,^19^ and the proliferation/differentiation of SSCs via ERK1/2–*Fos* mechanisms^20^, we examined the expression of selected GDNF signaling components. We observed that Cr(VI) decreased not only the transcriptional (mRNA) expression of receptor molecules such as glial cell line derived neurotrophic factor family receptor alpha 1 (*Gfra1*) and ret proto-oncogene (*Ret*) in a dose-dependent manner, but also the expression of downstream components such as v-myc myelocytomatosis viral related oncogene neuroblastoma derived homolog (*Mycn*) and FBJ osteosarcoma oncogene (*Fos*) (Fig. 6). In addition, Cr(VI) decreased the expression of two other proteins, P-AKT1 and P-ERK1/2, which are downstream of *Ret*, in a dose-dependent manner (Fig. 6). Our results indicate that Cr(VI) induced P-P53-dependent apoptosis in SSCs and disrupted the GDNF signaling pathway that is responsible for the differentiation and self-renewal of SSCs.

### Cr(VI) disrupts steroidogenesis in TM3 cells, affects expression of tight-junction signaling and cell receptor molecules, as well as impairs secretory functions in TM4 cells

**Figure 7:**
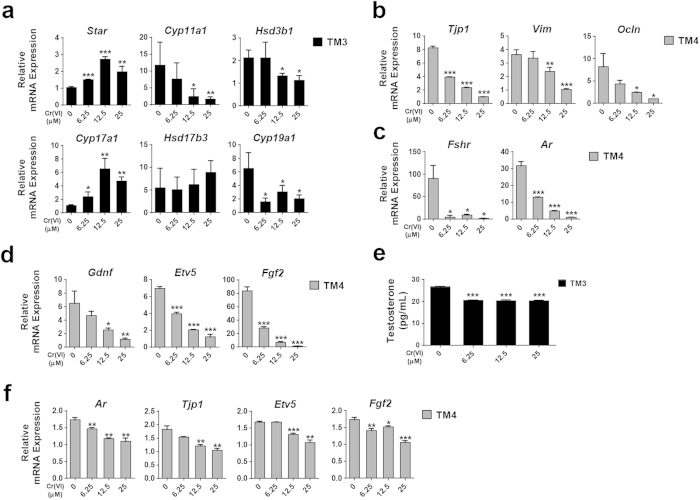
Effects of Cr(VI) on mRNA expression of steroidogenic enzymes in TM3 cells and tight junction signaling molecules, cell receptors, and factors responsible for SSC survival in TM4 cells. (**a**) Relative mRNA expression of *Star, Cyp11a1, Hsd3b1, Cyp17a1, Hsd17b3*, and *Cyp19a1* was analyzed by qRT-PCR in TM3 cells after treatment with different concentrations of Cr(VI) for 24 h. (**b**) Relative mRNA expression of *Tjp1, Vim*, and *Ocln* was analyzed by qRT-PCR in TM4 cells after treatment with different concentrations of Cr(VI) for 24 h. (**c**) Relative mRNA expression of *Fshr* and *Ar* was analyzed by qRT-PCR in TM4 cells after treatment with different concentrations of Cr(VI) for 24 h. (**d**) Relative mRNA expression of *Gdnf, Etv5*, and *Fgf2* was analyzed by qRT-PCR in TM4 cells after treatment with different concentrations of Cr(VI) for 24 h. (**e**) Tetstosterone release from TM3, Leydig cells into the media. TM3 cells were first treated with Cr(VI) for 24 h. Then, the media were removed and the cells were cultured further for 48 h in fresh meda without Cr(VI). After that the testosterone levels were measured in the media. (**f**) The relative mRNA expression of *Tjp1*, *Ar, Etv5*, and *Fgf2* was analyzed by qRT-PCR in TM4 cells after culture for 48 h with CM obtained from Cr(VI)-treated TM3 cells. All values are expressed as mean ± SEM. (*n = 3*). *P < 0.05, **P < 0.01 and ***P < 0.001 compared to the control.

We investigated the negative impact of Cr(VI) on TM3 and TM4 cell functions. In TM3 cells, we examined the effect of Cr(VI) on the transcriptional expression of testicular enzymes responsible for sex hormone synthesis, including cytochrome P450, family 11, subfamily a, polypeptide 1 (*Cyp11a1*), cytochrome P450, family 17, subfamily a, polypeptide 1 (*Cyp17a1*), cytochrome P450, family 19, subfamily a, polypeptide 1 (*Cyp19a1*), steroidogenic acute regulatory protein (*Star*), hydroxyl-delta-5-steroid dehydrogenase, 3 beta- and steroid delta-isomerase 1 (*Hsd3b1*), and hydroxysteroid (17-beta) dehydrogenase 3 (*Hsd17b3*) (Fig. 7). Exposure to Cr(VI) for 24 h significantly decreased the expression of *Cyp11a1* and *Hsd3b1* in a dose-dependent manner. Cr(VI) also decreased the expression of *Cyp19a1*. In contrast, the expression of *Cyp17a1* and *Star* significantly increased at all tested doses of Cr(VI) compared to the corresponding values of the control. The expression of *Hsd17b3* was not significantly affected by Cr(VI). Since the tight junctions of Sertoli cells form the blood-testis barrier^21^, we examined the effects of Cr(VI) on the transcriptional expression of tight-junction signaling molecules including tight junction protein 1 (*Tjp1*), vimentin (*Vim*), and occludin (*Ocln*) and cell receptors, such as follicle-stimulating hormone receptor (*Fshr*) and androgen receptor (*Ar*) that play a key role in the maturation and normal functioning of Sertoli cells^22^,^23^. We observed that exposure to increasing doses of Cr(VI) significantly decreased the expression of tigh-junction signaling molecules and cell receptors (Fig. 7). We also examined the effects of Cr(VI) on the transcriptional expression of glial cell line derived neurotrophic factor (*Gdnf*), ets variant gene 5 (*Etv5*), and fibroblast growth factor (*Fgf2*) in TM4 cells that are essential for the maintenance of SSCs (Fig. 7). Increasing doses of Cr(VI) significantly decreased the expression of these factors. Our results demonstrate that Cr(VI) impairs the physiological functions of TM3 and TM4 cells.

### Conditioned Medium (CM) from Cr(VI)-treated TM3 cells impairs TM4 cell functions

Finally we examined whether impaired steroidogenesis in TM3 cells induced by Cr(VI) toxicity had any direct effect on TM4 cell functions. We observed that Cr(VI)-treatment significantly decreased the testosterone release from the TM3 cells into the culture media (Fig. 7e). This culture media was mixed with fresh media at 1:1 ratio and used as conditioned media (CM) to culture the TM4, Sertoli cells for 48 h. We then measured the transcriptional expression of tight junction signaling molecules, cell receptors, and factors responsible for SSC survival in TM4 cells. We observed that increasing doses of conditional medium (CM) significantly decreased the expression of *Tjp1*, *Ar, Etv5* and *Fgf2* in TM4 cells (Fig. 7f). These results suggested that damage to TM3 cells might have deleterious effects on the physiological functions of TM4 cells.

## Discussion

This study aimed to determine the *in vitro* cytotoxic effects of Cr(VI) on mouse Leydig (TM3) cells, Sertoli (TM4) cells, and SSCs that have critical roles in spermatogenesis. We examined the effects of Cr(VI) on oxidative stress and apoptosis-related signaling mechanisms in TM3, TM4, and SSCs, cell proliferation/self-renewal mechanisms of SSCs, and the physiological functions of TM3 and TM4 cells. For this purpose, cells were treated with Cr(VI) at doses of 0, 3.125, 6.25, 12.5, 25, or 50 μM for 24 h; and then analyzed biochemically, and by flow cytometry, fluorescence microscopy, qRT-PCR, and immunoblotting.

To the best of our knowledge, this study is the first to report that the dose-dependent cytotoxic effects of Cr(VI) exposure in both male somatic cells and SSCs are mediated through apoptosis. Since somatic cells and SSCs play an important role in the process of spermatogenesis, damage to these cells has adverse effects on the production of healthy sperm cells. We investigated whether oxidative stress and mitochondrial dysfunction played a role in our current experimental model and observed that, after 4 h, exposure to Cr(VI) increased the production of ROS, which was maintained up to 24 h. In contrast, 24 h exposure to Cr(VI) decreased MMP in a dose-dependent manner. Treatment with a ROS inhibitor, NAC, abrogated the effects of Cr(VI). This demonstrates that oxidative stress and subsequent mitochondrial damage play a crucial role in Cr(VI)-induced cytotoxicity. We showed that the transcriptional expression of antioxidant enzymes that play a significant role in scavenging free radicals, including *Cat, Sod1, Sod2, Gpx1*, and *Gsta4*^24^,^25^,^26^,^27^,^28^,^29^ decreased with increasing doses of Cr(VI). Thus, the oxidative stress in the somatic cells and SSCs observed after exposure to Cr(VI) appears to be due to poor scavenging of free radicals by the antioxidant enzymes. These results are supported by those of previous reports demonstrating that Cr(VI)-induced oxidative stress via the suppression of antioxidant enzymes plays a major role in male infertility^16^,^30^,^31^. The over-expression of *Gsta1* (seen with lower doses of Cr(VI) in SSCs and all doses in somatic cells) might protect the cells against Cr(VI)-induced oxidative stress.

The induction of the mitochondria-dependent (intrinsic) pathway of apoptosis in the male somatic cells observed after Cr(VI) exposure, was confirmed by the decreased BCL2/BAX protein ratio, increased expression of cytosolic CYCS, and cleavage of CASP9, CASP3, and PARP proteins. Cell viability depends on the balance between survival and pro-apoptotic signaling, regulated by the AKT1, MAPK, and P53 pathways^32^,^33^. AKT1 and ERK1/2 signaling pathways are associated with cell proliferation and survival^34^,^35^, while ROS produced by several toxicants, including Cr(VI), serve as second messengers to activate pro-apoptotic kinases, such as JNK1/2 and P38^7^,^36^,^37^,^38^,^39^,^40^,^41^,^42^,^43^. Cr(VI)-induced DNA damage and oxidative stress also promoted the activation of P53 leading to intrinsic apoptosis^33^,^44^,^45^,^46^,^47^. We showed that, in male somatic cells, exposure to Cr(VI) increased the phosphorylation of JNK1/2 and P38 in a dose-dependent manner, while the phosphorylation of ERK1/2 decreased (down-regulation was higher in TM4 cells). The expression levels of P-AKT 1 were not altered up to the IC_50_ dose level of Cr(VI). At a concentration of 25 μM, however, Cr(VI), decreased the expression of P-AKT1, by only 20% in TM3 cells, but a 40% reduction was observed in TM4 cells. Cr(VI) also increased P-P53 expression in a dose-dependent manner (up-regulation was more pronounced in TM3 cells). These cell-specific differences in the regulation of P-AKT1, P-ERK1/2, and P-P53 proteins are probably at least partially responsible for the differential sensitivity of TM3 and TM4 cells to Cr(VI).

The increased phosphorylation of P53 and cleavage of CASP9, CASP3, and PARP proteins showed that Cr(VI) induced intrinsic apoptosis in SSCs at concentrations up to 12.5 μM, but a higher concentration (25 μM) decreased apoptosis. We therefore investigated other factors, apart from apoptosis, that could also be involved in Cr(VI)-induced cytotoxicity. In SSCs, GDNF plays two major roles, regulating the self-renewal/maintenance via AKT1–*Mycn* signaling pathways^18^,^19^ and the proliferation/differentiation via ERK1/2–*Fos* pathways^20^. We demonstrated that Cr(VI) not only affected the self-renewal/maintenance pathway but also the proliferation/differentiation pathways in SSCs in a dose-dependent manner. Our results demonstrated that Cr(VI) (i) at 6.25 μM (IC_50_), induced cytotoxicity mainly through P53-dependent apoptosis; (ii) at 12.5 μM, increased apoptosis and disrupted GDNF signaling pathway; and (iii) at 25 μM, decreased apoptosis (like 6.25 μM) and further disrupted GDNF signaling pathway (compared to 12.5 μM). In conclusion, the loss of cell viability observed at higher doses of Cr(VI), involves the apoptosis pathways and impairment of the GDNF signaling pathway.

Cr(VI) toxicity has been reported to disrupt steroidogenesis and decrease serum testosterone levels and 3β-HSD enzyme activity in male rats^48^,^49^,^50^. We also observed that Cr(VI)-treatment significantly decreased the testosterone release from the TM3 cells into the culture media. Besides, Cr(VI) exposure in TM3 cells significantly decreased transcriptional expression of *Cyp11a1* (a rate-limiting enzyme in steroidogenesis that converts cholesterol to pregnenolone in mitochondria) and *Hsd3b1* (that converts pregnenolone to progesterone in the endoplasmic reticulum), without significant alteration in the levels of *Hsd17b3*. In contrast, Cr(VI) increased the expression of *Cyp17a1* and *Star*, possibly through a negative feedback compensatory mechanism counterbalancing the Cr(VI)-induced inhibition of *Cyp11a1* and *Hsd3b1*. Steroidogenesis is a complex multi-enzyme process wherein steroid hormones are produced from cholesterol. A number of researchers have shown that impairment of steroidogenesis by environmental toxicants does not require the inhibition of all the steroidogenic enzyme genes^51^,^52^,^53^. In the present study, therefore, Cr(VI) impaired steroidogenesis, at least to some extent, by inhibiting *Cyp11a1* and *Hsd3b1* in TM3 cells. Cr(VI) also decreased the expression of *Cyp19a1*, which is required for the conversion of androgens into estrogens. In TM4 cells, Cr(VI) decreased the transcriptional expression of the tight-junction signaling molecules, *Tjp1, Vim*, and *Ocln*, which play a key role in forming the blood-testis barrier^21^. These results are supported by those of the previous report where the authors showed that Cr(VI) altered the Sertoli cell barrier, which might affect the blood-testis barrier^54^.

Cr(VI) also decreased the transcriptional expression of the cell receptors, *Fshr* and *Ar*, which play a key role in the maturation and normal functioning of Sertoli cells^22^,^23^ and those of some secreted molecules, including *Gdnf, Etv5*, and *Fgf2*, which are essential for the maintenance of SSCs^55^ within the stem cell niche. Our results suggest that Cr(VI)-toxicity may also affect the maintenance of SSCs *in vivo*. Thus, Cr(VI)-induced impairment of the physiological functions of TM3 and TM4 cells could directly affect normal spermatogenesis.

**Figure 8:**
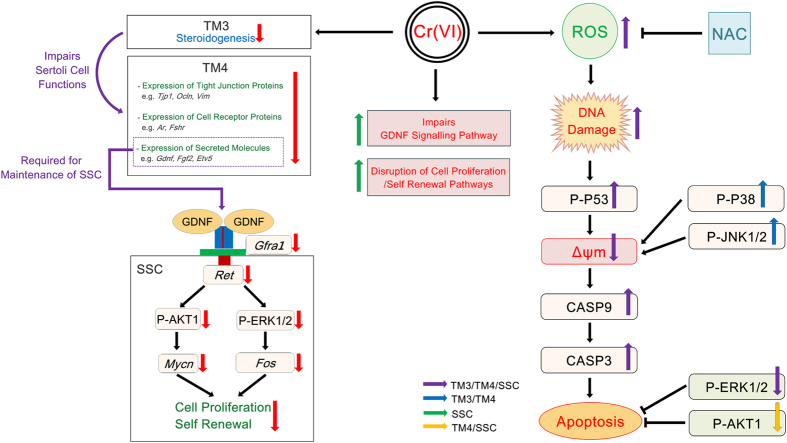
Schematic diagram of Cr(VI) induced cytotoxicity in male somatic cells and SSCs. Effect of Cr(VI) on the pro-apoptotic and pro-survival signaling pathways, physiological functions of somatic cells, cell proliferation/self-renewal pathways in SSCs.

In conclusion, Cr(VI) induced mitochondria-dependent apoptosis in male somatic cells and SSCs, possibly through the induction of oxidative stress and DNA damage (Fig. 8). The cell-specific pattern of regulation of P-AKT1, P-ERK1/2, and P-P53 proteins is probably responsible, at least in part, for the differential sensitivity of TM3 and TM4 cells to Cr(VI). Cr(VI) also disrupted the differentiation and self-renewal mechanisms of SSCs and impaired the physiological functions of TM3 and TM4 cells. To the best of our knowledge, our study is the first to uncover the underlying signaling mechanisms of Cr(VI)-induced cytotoxicity and apoptosis in male somatic cells and SSCs. Our experimental findings could provide a basis for the development of improved antagonists of Cr(VI) to combat Cr(VI)-induced reproductive toxicity.

## Methods

### Materials

K_2_Cr_2_O_7_ (molecular weight, 294.18) was purchased from Duksan pure chemical Company Limited, (Ansan, South Korea). Penicillin-streptomycin solution, trypsin-EDTA solution, DMEM, and 1% antibiotic-antimycotic solution were obtained from Life Technologies GIBCO (Grand Island, NY, USA). Fetal bovine serum (FBS) and an *in vitro* toxicology assay kit were purchased from Sigma-Aldrich (St. Louis, MO). The antibodies used for immunoblotting were against phospho P53, phospho ERK1/2, total ERK1/2, total AKT1 (Cell Signaling Technology, Beverly, MA), phospho P38 (Santa Cruz Biotechnology Inc., Santa Cruz, CA), phospho AKT1, phospho JNK1/2, total P53, total JNK1/2, total P38, BAX, BCL2, CASP9, CASP3, PARP, beta-actin (Abcam, Cambridge, MA), and cytochrome c (ENZO Diagnostics Inc., Farmingdale, NY).

### Cell culture and treatment

Mouse Leydig and Sertoli (TM3, TM4) cells were obtained from the Korean Cell Line Bank (TM3, KCLB 21714; TM4, KCLB 21715). The cells were cultured in DMEM, supplemented with 10% FBS and 100 U/mL penicillin-streptomycin, in a humidified incubator maintained at 37 °C, in the presence of 5% CO_2_. For *in vitro* cytotoxicity experiments, cells were treated with different concentrations of K_2_Cr_2_O_7_ (0, 3.125, 6.25, 12.5, 25, and 50 μM) for 24 h in DMEM, supplemented with 1% FBS and 100 U/mL penicillin-streptomycin. The spermatogonial stem cells were isolated from 1-week-old ICR mice testis^56^,^57^. Testes of 1-week-old males were collected in PBS. After removing the tunica albuginea, the seminiferous tubules were treated with collagenase and trypsin to obtain a single-cell suspension. CD90-positive testicular cells were enriched by MACS (microbeads, Miltenyi Biotec, Bergisch Gladbach, Germany). The CD90-positive cells were cultured on mitomycin C-inactivated MEF feeder cells, in a medium containing 1% FBS (HyClone, Thermo Scientific, MA, USA), 1 × penicillin/streptomycin (GIBCO, Grand Island, NY, USA), 1 × NEAA (Sigma), 0.1 mM β-ME (GIBCO, Grand Island, NY, USA), 25 μg/ml insulin (Sigma-Aldrich, St. Louis, MO), 100 μg/ml transferrin (Sigma-Aldrich, St. Louis, MO), 0.19 μM progesterone (Sigma-Aldrich, St. Louis, MO), 60 μM putrescine (Sigma-Aldrich, St. Louis, MO), 0.03 μM sodium selenite (Sigma-Aldrich, St. Louis, MO), 50 μg/ml BSA (Sigma-Aldrich, St. Louis, MO), 10 ng/ml GDNF (R&D systems, MN, USA), 20 ng/ml EGF (R&D systems, MN, USA), 10 ng/ml bFGF (R&D systems, MN, USA), 10^3^ units/ml LIF (ESGRO Millipore, Darmstadt, Germany). This study was carried out in strict accordance with the recommendations in the Guide for the Care and Use of the Konkuk University Animal Care and Experimentation Community. All experimental protocols were approved by the Committee on the Ethics of Animal Experiments of the Konkuk University (IACUC approval number: KU11035).

### Cell viability and cytotoxicity assay

Cells were seeded (5 × 10^5^ cells/well) onto 96-well, flat bottom culture plates and incubated for 24 h at 37 °C in a 5% CO_2_ incubator. The used medium was replaced by fresh DMEM containing 1% FBS. Cells were then treated with different concentrations of K_2_Cr_2_O_7_ (0, 3.125, 6.25, 12.5, 25 and 50 μM) for 24 h in a humidified incubator at 37 °C in the presence of 5% CO_2_. A cell viability assay was performed using the Cell Counting Kit-8 (CCK-8, Dojindo Laboratories, Kumamoto, Japan). The absorbance was read at a wavelength of 450 nm, using a microtiter plate reader (Multiskan FC, Thermo Fisher Scientific Inc., Waltham, MA, USA). Cytotoxicity was assessed by LDH assay in the supernatant medium using an LDH Cytotoxicity Detection kit (Takara Bio Inc., Tokyo, Japan), according to the protocol of the manufacture, by measuring the absorbance at 490 nm using a microplate reader.

### TUNEL/TMR red assay

Cells were grown on glass slides and, after reaching 80% confluency, were treated with K_2_Cr_2_O_7_. The cells were washed with PBS, fixed with 4% paraformaldehyde, washed again, and incubated with 0.1% Triton X-100. TUNEL analysis was performed to measure the degree of cellular apoptosis, using an *in situ* Cell Death Detection Kit, TMR red (Roche Diagnostics, Indianapolis, IN) according to the manufacturer’s instructions.

### Assessment of apoptotic cell populations

Cell death was analyzed using FITC conjugated Annexin V and propidium iodide with an Apoptosis detection kit (Life Technologies, Oregon, USA) according to the manufacturer’s instructions. Cells were characterized using FACSCalibur and the data were analyzed by Cell Quest software. Each analysis recorded 10^4^ events.

### Caspase 3 enzyme activity measurement

Caspase 3 enzyme activity was measured using a fluorometric assay kit (Abcam, Cambridge, MA), following the manufacturer’s instructions.

### Intracellular ROS measurement

Cells were incubated with 10 μM 2′, 7′-dichlorodihydrofluoresceindiacetate (H_2_-DCFDA) (Sigma-Aldrich, St. Louis, MO) in a humidified incubator at 37 °C for 30 min. Cells were then washed with phosphate-buffered saline (PBS) and resuspended in PBS. Fluorescence emission was measured at excitation and emission wavelengths of 488 nm and 515 nm, respectively, using a Gemini EM (SpectraMAX, Molecular Devices, Sunnyvale, CA).

### MMP measurement

MMP was evaluated using the cationic fluorescent indicator JC-1 (Molecular Probes Eugene, OR, USA). JC1-aggregates in intact mitochondria give red fluorescence with an emission at 583 nm and JC1-monomers in the cytoplasm give green fluorescence with an emission at 525 nm and an excitation wavelength at 488 nm. Cells were incubated with 10 μM JC-1 at 37 °C for 15 min, washed with PBS, then resuspended in PBS, and the fluorescence intensity was measured. MMP was expressed as the ratio of the fluorescence intensity of the JC1 aggregates to JC1 monomers.

### Preparation of CM from Cr(VI)-treated TM3 cells and culture of TM4 cells with CM

TM3, Leydig cells were divided into four groups and treated with different concentrations of K_2_Cr_2_O_7_ (0, 6.25, 12.5 and 25 μM) for 24 h in a humidified incubator at 37 °C in the presence of 5% CO_2_. Then, the medium were removed, the cells were washed with PBS, and cultured for 48 h in fresh medium without Cr(VI). The supernatant from each group was harvested, centrifuged to remove debris and mixed (1:1) with fresh medium to prepare conditioned medium. This CM was used to culture the TM4, Sertoli cells for 48 h and after that, we measured the mRNA expression of tight-junction signaling molecules, cell receptors, and factors responsible for SSC survival in TM4 cells.

### Measurement of testosterone level

TM3, Leydig cells were divided into four groups and treated with different concentrations of K_2_Cr_2_O_7_ (0, 6.25, 12.5 and 25 μM) for 24 h in a humidified incubator at 37 °C in the presence of 5% CO_2_. Then, the medium were removed, the cells were washed with PBS, and cultured for 48 h in fresh medium without Cr(VI). The supernatant from each group was harvested, centrifuged to remove debris and the testosterone levels were measured using a spectrophotometric ELISA kit (ENZO Diagnostics Inc., Farmingdale, NY), following the manufacturer’s instructions.

### Gene expression analysis

mRNA from the treated cells was extracted using an RNeasy Mini Kit (Qiagen) and cDNA was synthesized using a QuantiTect Reverse Transcription Kit (Qiagen) in a final volume of 20 μL, according to the manufacturer’s instructions. All gene transcripts were measured in triplicate by qRT-PCR on a LightCycler apparatus, using LightCycler^®^ FastStart DNA Master SYBR Green I with an AB Applied Biosystems machine. The primer sequences for each gene are shown in Table S1. The relative quantification of gene expression was analyzed by the 2-ddCt method^58^. In all experiments, *Gapdh* mRNA was used as an internal standard.

### Immunoblotting

Cells were lysed in radioimmunoprecipitation (RIPA) lysis buffer containing protease and phosphatase inhibitors. Equal amounts of protein were resolved by 10% sodium dodecyl sulfate-polyacrylamide gel electrophoresis (SDS-PAGE) and proteins were electrophoretically transferred to PVDF membranes. Membranes were blocked at room temperature with 5% non-fat dry milk for 2 h to prevent non-specific binding, and then incubated with primary antibodies overnight at 4 °C. Immunoreactivity was detected through sequential incubation with horseradish peroxidase-conjugated secondary antibodies and enhanced chemiluminescence reagents.

### Statistical analysis

All the experiments were performed at least in triplicate, and statistical analyses were performed by one-way analysis of variance (ANOVA) followed by Dunnett’s *t*-test to determine the significance of difference between the treatment and control groups. Statistical tests were performed using Stat View version 5.0 (SAS institute, Cary, NC, USA). The level of significance was set at *p < 0.05, **p < 0.01 and ***p < 0.001 compared to the control.

## Additional Information

**How to cite this article**: Das, J. *et al.* Hexavalent chromium induces apoptosis in male somatic and spermatogonial stem cells via redox imbalance. *Sci. Rep.* **5**, 13921; doi: 10.1038/srep13921 (2015).

## Supplementary Material

Supplementary Information
