# Supplementary material for: Hexavalent chromium induces apoptosis in male somatic and spermatogonial stem cells via redox imbalance
Source: Sci Rep. 2015 Sep 10;5:13921. doi: 10.1038/srep13921 (PMC4564811; doi:10.1038/srep13921)

**Hexavalent chromium induces apoptosis in male somatic and spermatogonial stem cells via redox imbalance**

Joydeep Das^1^, Min-Hee Kang^1^, Eunsu Kim^1^, Deug-Nam Kwon^1^, Yun-Jung Choi^1^, Jin-Hoi Kim^1^*

^1^Department of Animal Biotechnology, College of Animal Bioscience and Biotechnology/Animal Resources Research Center, Konkuk University, Seoul 143-701, South Korea

**Corresponding author:**

**Jin-Hoi Kim:** [jhkim541@konkuk.ac.kr](mailto:jhkim541@konkuk.ac.kr)

**Parmanent address:** Department of Animal Biotechnology, College of Animal Bioscience and Biotechnology/Animal Resources Research Center, Konkuk University, Seoul 143-701, South Korea

**Supplementary Table 1**

| **Primer name** | **Primer sequence** | **Annealing** |
| --- | --- | --- |
| ***Cat*** | F: TTGACAGAGAGCGGATTCCT  R: TCTGGTGATATCGTGGGTGA | 55°C |
| ***Sod1*** | F: CCAGTGCAGGACCTCATTTT  R: AGTCACATTGCCCAGGTCTC | 55°C |
| ***Sod2*** | F: CCAAAGGAGAGTTGCTGGAG  R: GAACCTTGGACTCCCACAGA | 55°C |
| ***Gpx1*** | F: GGTTCGAGCCCAATTTTACA  R: CATTCCGCAGGAAGGTAAAG | 55°C |
| ***Gsta1*** | F: CGCCACCAAATATGACCTCT  R: TTGCCCAATCATTTCAGTCA | 55°C |
| ***Gsta4*** | F: AGCCATTTTGATGGTGGAAG  R: CGGGTTGCAGGAACTTCTTA | 55°C |
| ***Gfra1*** | F: GTGGCAATGACCTGGAAGAT  R: ATTGCCAAAGGCTTGAATTG | 55°C |
| ***Ret*** | F: GCTGCATGAGAATGACTGGA  R: CTGTTCCCAGGAACTGTGGT | 55°C |
| ***Mycn*** | F: GCGGTAACCACTTTCACGAT  R: AGTTGTGCTGCTGATGGATG | 55°C |
| ***Fos*** | F: ATCCTTGGAGCCAGTCAAGA  R: ATGATGCCGGAAACAAGAAG | 55°C |
| ***Star*** | F: GCAGCAGGCAACCTGGTG  R: TGATTGTCTTCGGCAGCC | 58°C |
| ***Cyp11a1*** | F: CGATACTCTTCTCATGCGAG  R: CTTTCTTCCAGGCATCTGAAC | 58°C |
| ***Hsd3b1*** | F: CTGAATGTTACTGGCAAATTCTC  R: TGTAAAATGGACGCAGCAGGAA | 58°C |
| ***Cyp17a1*** | F: TTTTGGCCCAAGTCAAAGAC  R: CCCTTCTTCACGAGCACTTC | 55°C |
| ***Hsd17b3*** | F: GTTCTCGCAGCACCTTTTTC  R: ACAATCTTCACACCGCTTCC | 58°C |
| ***Cyp19a1*** | F: CACCCTTCCAAGTGACAGGA  R: AAAAAAGTAAAGTTCTATGGGAA | 58°C |
| ***Tjp1*** | F: CTCTGGTGGAAGAGATAATCCTCA  R: GTTTTTCCCACTCTTCCTTAGCTG | 60°C |
| ***Vim*** | F: TTTGCCAACTACATCGACAAGGT  R: CATCTCCTCCTGCAATTTCTCTC | 60°C |
| ***Ocln*** | F: GCGGAAAGAGTTGACAGTCC  R: GGCACCAGAGGTGTTGACTT | 55°C |
| ***Fshr*** | F: CATCACTGGGAACACCACAG  R: ATCCCAATGCAAAGATCAGC | 55°C |
| ***Ar*** | F: TGCCTCCGAAGTGTGGTATC  R: CCGTAGTGACAGCCAGAAGC | 55°C |
| ***Gdnf*** | F: ATTCAAGCCACCATTAAAAGACTG  R: GAAAGATCAGTTCCTCCTTGGTTT | 60°C |
| ***Etv5*** | F: CTTGGTTAGCTGAAGCACAAGTTC  R: TTCTCCATACTTAGCACCAAGAGC | 60°C |
| ***Fgf2*** | F: AAGCGGCTCTACTGCAAGAAC  R: CTTCATAGCAAGGTACCGGTT | 60°C |
| ***Gapdh***  ***Fas*** | F: AGGTCGGTGTGAACGGATTTG  R: TGTAGACCATGTAGTTGAGGTCA  **F: AATGAATGCCAACTGTATT**  **R: CACTGAGTATCAACTACCA** | 55°C  53°C |
| ***Fasl*** | **F: TTGTACTTCGTGTATTCCA**  **R: GTTCCTCATATAGACCTTGT** | 53°C |
| ***Casp8*** | **F: GAGTTGCCACCTTCAGTT**  **R: TCACTGTCTTGTTCTCTTGG** | 53°C |

**Supplementary Figure legends**

**Figure 1: Effect of N-acetylcysteine on Cr(VI)-induced cytotoxicity of in male somatic cells and SSCs**

**a.** Cell viability relative to the control (100%) in the mouse TM3 cells. **b.** Cell viability relative to the control (100%) in the mouse TM4 cells. **c.** Cell viability relative to the control (100%) in the mouse SSCs. Cells were pre-treated with 5 mM NAC for 1 h and then exposed to different concentrations of Cr(VI) in μM for 24 h. All values were expressed as mean ± S.E.M. (*n = 3*). ^#^*P* < 0.05 compared with the Cr(VI)-treated groups.

**Figure 2: Effect of N-acetylcysteine on Cr(VI)-induced ROS formation in male somatic cells and SSCs**

**a-c.** Representative bright field and fluorescent images of TM3, TM4 cells and SSCs respectively, after staining with cationic fluorescent dye, 2',7'-dichlorodihydrofluorescein diacetate (H2-DCFDA). Cells were first grown on glass slide, pre-treated with 5 mM NAC for 1 h and then treated with 12.5 μM Cr(VI) for 24 h followed by incubation with 10 µM H_2_-DCFDA at 37°C for 30 min. Then cells were washed with PBS, mounted with fluorescent medium, covered with glass cover slip and observed under a fluorescent microscope.

**Figure 3: Effect of N-acetylcysteine on Cr(VI)-induced MMP loss in male somatic cells and SSCs**

**a-c.** Representative fluorescent images of TM3, TM4 cells and SSCs respectively, after staining with fluorescent dye, JC1. Cells were first grown on glass slide, pre-treated with 5 mM NAC for 1 h and then treated with 12.5 μM Cr(VI) for 24 h followed by incubation with 10 µM JC1 at 37°C for 15 min. Then cells were washed with PBS, mounted with fluorescent medium, covered with glass cover slip and observed under a fluorescent microscope.

**Figure 4: Effects of Cr(VI) on mRNA expression of genes related to extrinsic apoptosis in TM4 cells**

The relative mRNA expression of *Fasl, Fas and Casp8* was analyzed by qRT-PCR in TM3, TM4 cells and SSCs after treatment with different concentrations of Cr(VI) for 24 h. All values are expressed as mean ± S.E.M. (*n = 3*). **P* < 0.05, ***P* < 0.01 and ****P* < 0.001 compared to the control.

**
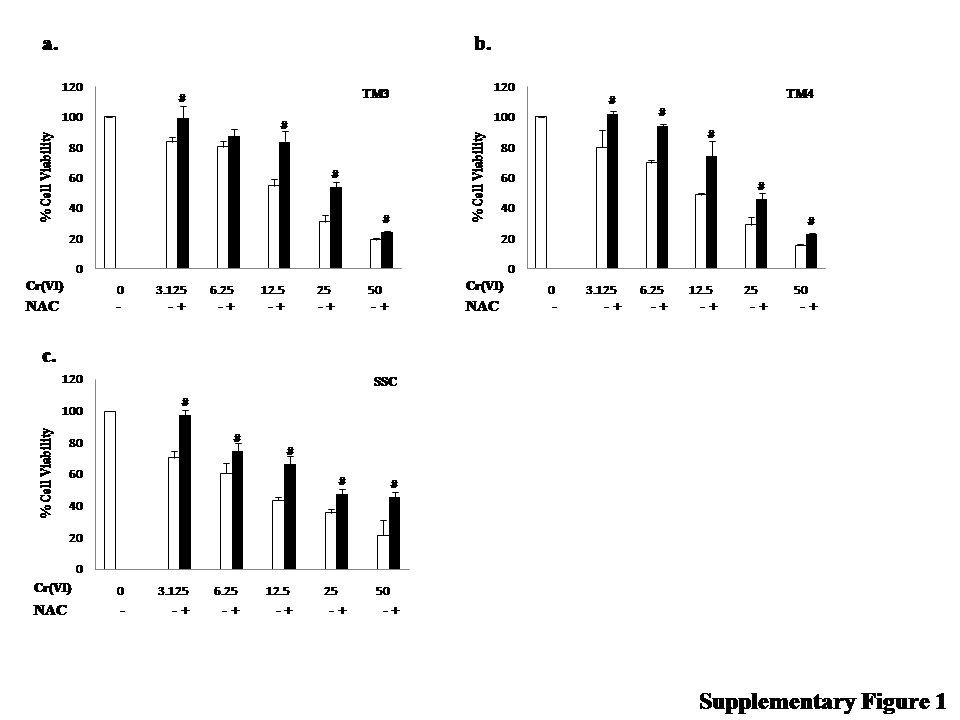
**

**
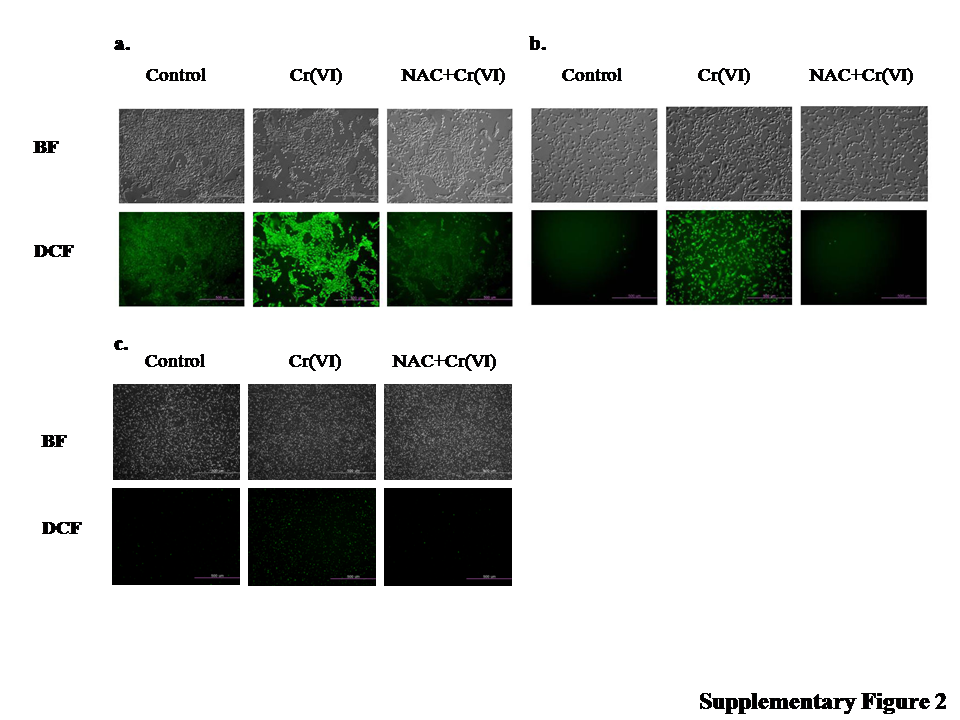
**


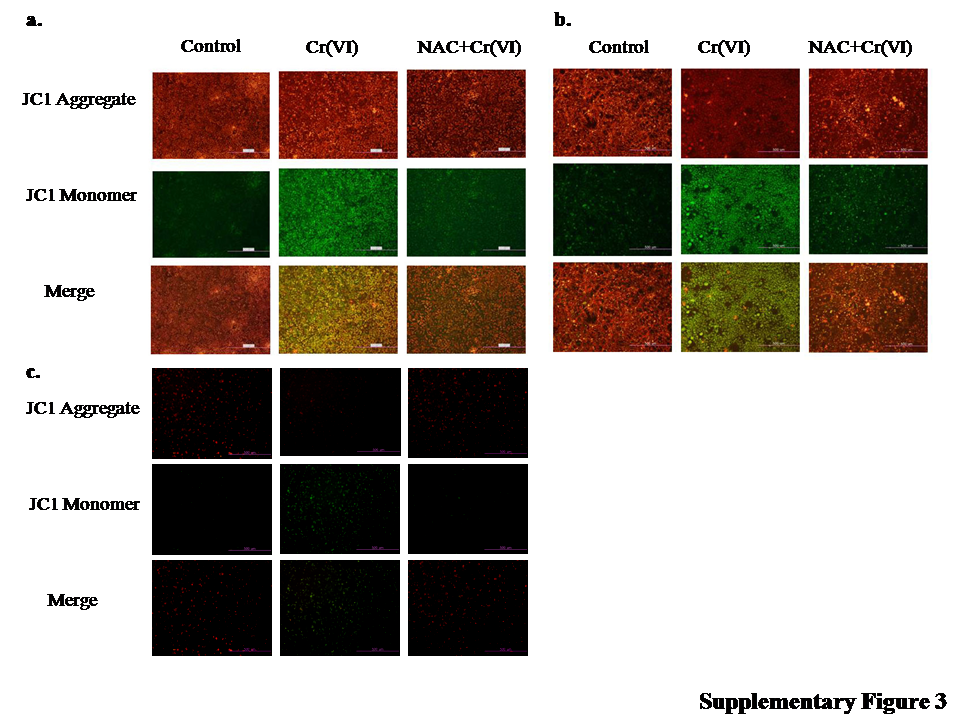


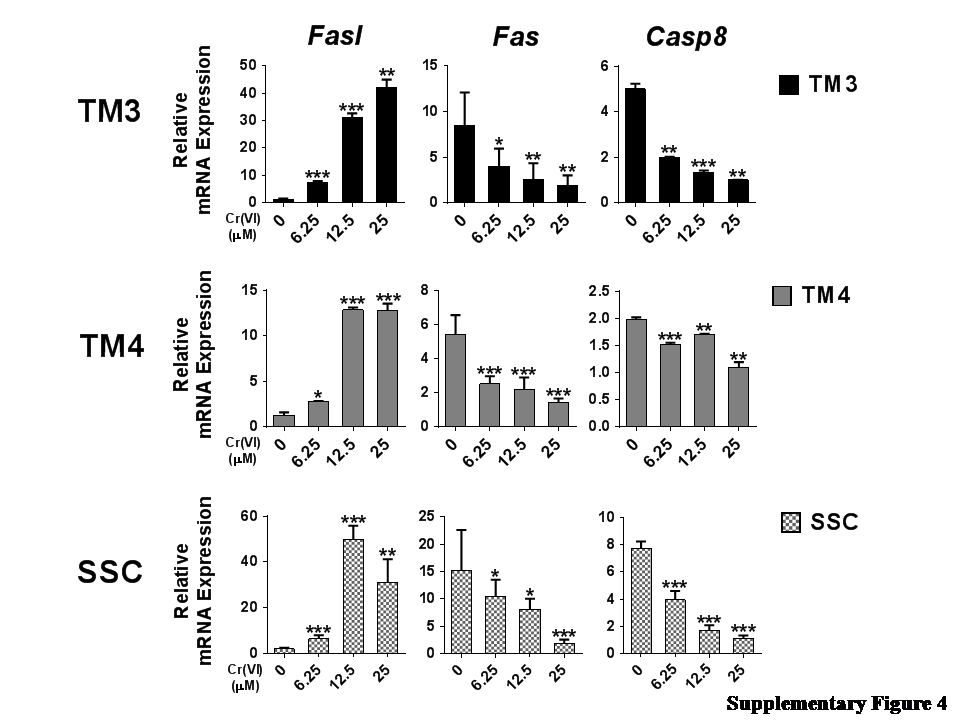

Supplement: Supplementary Information [file srep13921-s1.doc]
